# A Case Report on the Diagnostic Challenge of Mandibular Osteolipoma

**DOI:** 10.7759/cureus.113071

**Published:** 2026-07-21

**Authors:** Bhumika V Bhatia, Deepa D Achath, Bhakti Patil, Pooja Rathod, Prathmesh Patil

**Affiliations:** 1 Oral Medicine and Radiology, Dr. G. D. Pol Foundation, Y.M.T. Dental College and Hospital, Navi Mumbai, IND; 2 Oral and Maxillofacial Surgery, Dr. G. D. Pol Foundation, Y.M.T. Dental College and Hospital, Navi Mumbai, IND; 3 Oral Pathology and Microbiology, Dr. G. D. Pol Foundation, Y.M.T. Dental College and Hospital, Navi Mumbai, IND

**Keywords:** lipoma variant, mandible, oral cavity, osseous metaplasia, osteolipoma

## Abstract

Osteolipoma is an extremely rare histological variant of lipoma characterized by the presence of mature adipose tissue admixed with well-formed lamellar bone. Its occurrence in the oral cavity is exceptional, often posing a diagnostic challenge due to its nonspecific clinical and radiographic features. We report a rare case of osteolipoma in a 40-year-old male who presented with a slow-growing, painless swelling on the right side of the mandible of six months' duration. Clinical examination revealed a firm, mobile, non-tender mass causing facial asymmetry and intraoral vestibular obliteration. Radiographic evaluation, including mandibular lateral topographic radiograph and cone beam computed tomography, demonstrated a well-defined mixed-density lesion with internal calcifications, initially suggestive of a peripheral ossifying fibroma. Complete surgical excision was performed. Histopathological examination revealed an encapsulated lesion composed of lobules of mature adipocytes interspersed with trabeculae of mature bone, confirming the diagnosis of osteolipoma. The patient was recalled one week postoperatively and placed on periodic clinical and radiographic follow-up at three months interval, and no recurrence was noted. Osteolipoma of the oral cavity is a rare benign neoplasm with excellent prognosis following complete surgical excision. Due to its rarity and resemblance to other calcified soft-tissue lesions, histopathological examination remains essential for definitive diagnosis. Reporting such cases contributes to a better understanding of the clinicopathological spectrum and biological behavior of this uncommon entity.

## Introduction

A lipoma is a benign mesenchymal neoplasm composed of mature adipocytes and is among the most common soft tissue tumours of the body. However, its occurrence in the oral cavity is relatively rare, accounting for approximately 1-4% of all benign oral soft tissue tumours [1,2]. Based on histopathological features, several variants of oral lipoma have been described, including fibrolipoma, angiolipoma, spindle cell lipoma, chondrolipoma, and osteolipoma [3].

Osteolipoma is an extremely rare histological variant of lipoma characterized by the presence of mature adipose tissue admixed with well-formed lamellar bone [4]. It may arise as a primary lesion or develop secondary to osseous metaplasia within a pre-existing lipoma. Although its exact pathogenesis remains unclear, proposed mechanisms include metaplastic transformation of fibroblasts into osteoblasts induced by chronic irritation, ischemia, or trauma, as well as differentiation from pluripotent mesenchymal stem cells [5,6].

In the oral cavity, osteolipoma is exceptionally uncommon, with only sporadic cases reported. To the best of our knowledge of the currently available English-language literature, approximately 10 cases involving the mandible have been reported to date, making the present case an exceptionally uncommon entity.

Other intraoral sites include the tongue, buccal mucosa, floor of the mouth, palate, and gingiva [4,7]. Clinically, ostelipomas typically present as slow-growing, painless, well-circumscribed masses, often mimicking conventional lipomas or other benign soft tissue tumours. Radiographic examination may occasionally reveal areas of calcification or ossification, which can assist in preoperative diagnosis [6].

Histopathological examination remains the gold standard for definitive diagnosis, revealing lobules of mature adipocytes interspersed with trabeculae of mature bone without cellular atypia [3,5]. Complete surgical excision is the treatment of choice, and recurrence is exceedingly rare, with an excellent prognosis [7]. Due to its rarity and potential diagnostic dilemma, documentation of additional cases of oral osteolipoma is important to broaden existing knowledge regarding its clinicopathological characteristics and biological behaviour.

The present case report describes a rare occurrence of osteolipoma of the oral cavity, emphasizing its clinical and radiological presentation, histopathological features, and management.

## Case presentation

A 40-year-old male patient presented to the department of Oral Medicine and Radiology with a painless swelling on the right side of the lower jaw. The patient was apparently alright six months back when he noticed a small swelling on the lower border of the mandible. In the subsequent months, he noticed the progressive enlargement of the swelling, and hence he visited a general hospital. He was then referred to the dental hospital for management of the same.

On examination, there was a gross facial asymmetry due to the diffuse swelling (approximately 3 x 3 cm in size) on the right lower jaw, which was nontender, hard in consistency, mobile, non-compressible, and non-fluctuant (Figure 1). It extended anteroposteriorly from the corner of the lip to a perpendicular from the outer canthus of the eye. Superoinferiorly from approximately 1 cm below the lip to the lower border of the mandible without expanding it. The skin over the swelling was slightly stretched and afebrile. The patient gave no history of paraesthesia.

**Figure 1:**
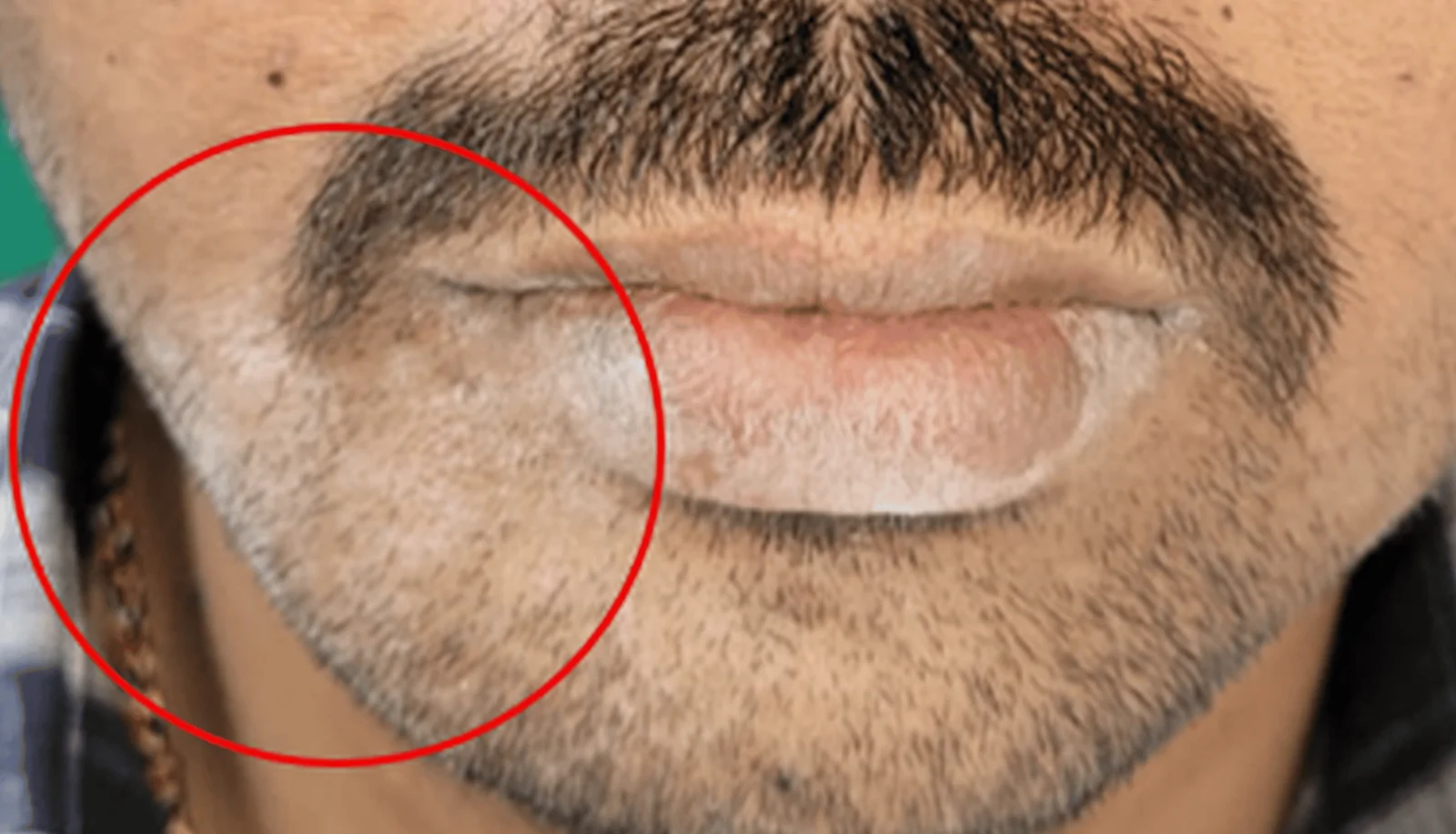
Gross facial asymmetry due to diffuse swelling on the right lower jaw

Intraorally, there was obliteration of the right buccal vestibule with a firm to hard swelling extending from 44 to 47, which was nontender and mobile (Figure 2). The overlying mucosa was similar to the adjacent healthy mucosa with no bleeding or ulceration. There were grade I stains, calculus, and root pieces in relation to 16, proximal caries with 26.

**Figure 2:**
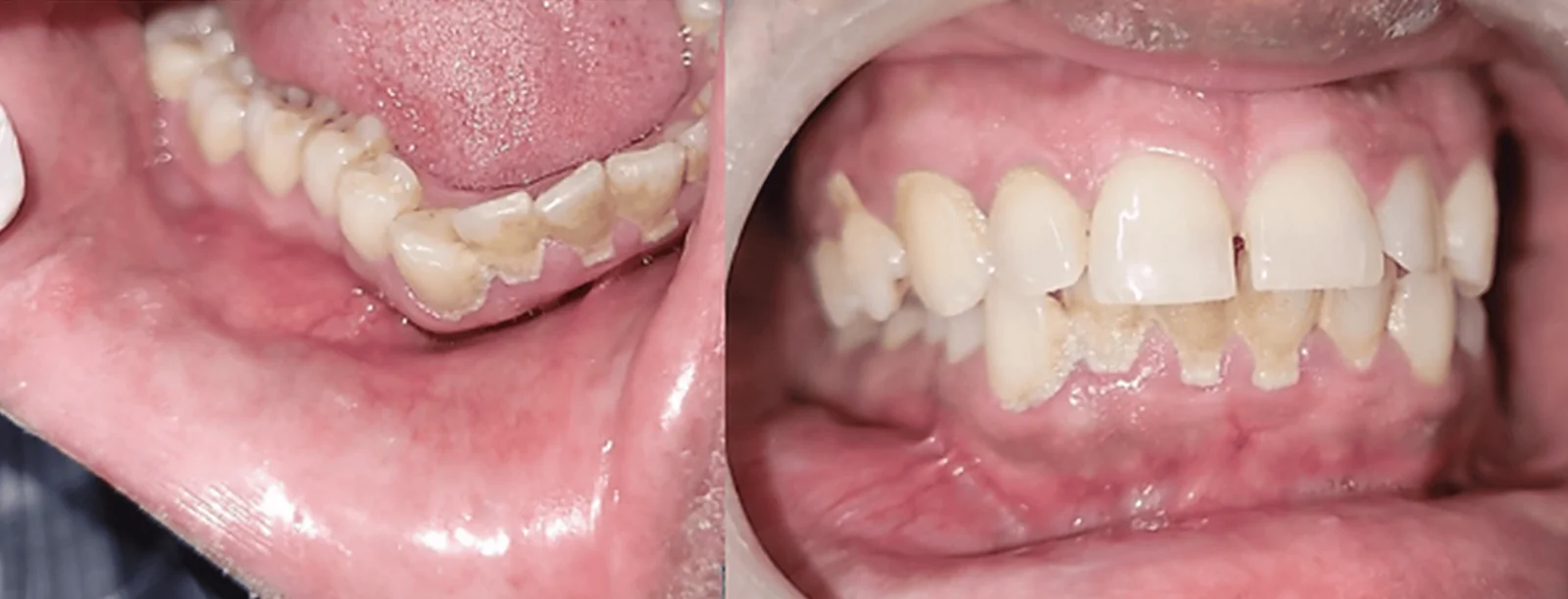
Obliteration of the right buccal vestibule due to a swelling extending from 44 to 47 region

On the basis of the clinical findings, we arrived at a provisional diagnosis of a benign non-odontogenic lesion on the lower right mandible. As part of the investigations, a mandibular lateral topographic radiograph and a cone beam computed tomography (CBCT) scan were taken, followed by a biopsy for confirmatory diagnosis.

Mandibular lateral topographic occlusal radiograph of the swelling presented with a well-defined mixed lesion with thin bony septae in a dense skeletal framework, which was not uniformly corticated (Figure 3).

**Figure 3:**
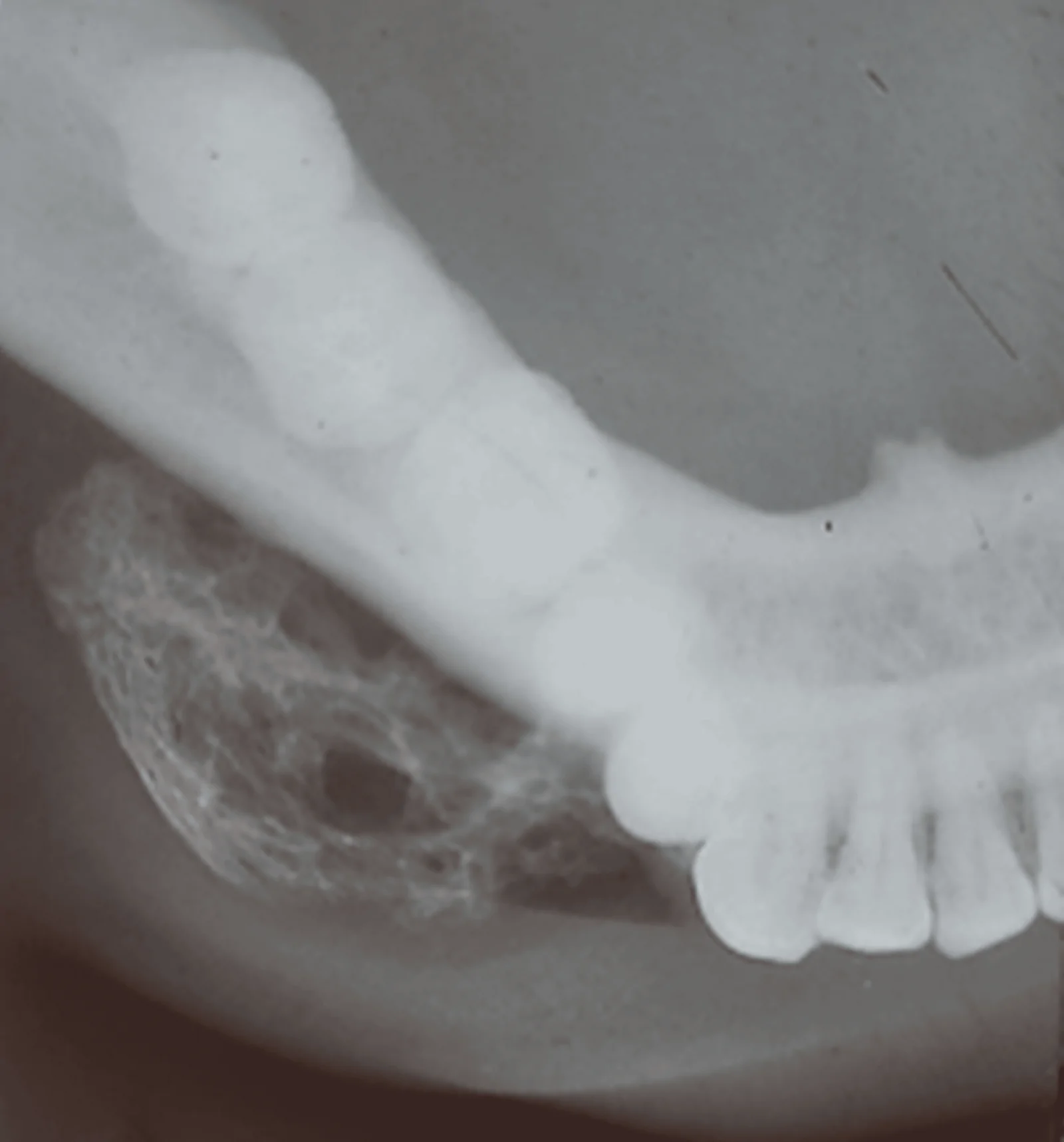
Mandibular occlusal radiograph demonstrating a well-defined mixed lesion with thin bony septa within a dense osseous framework

The CBCT scan revealed a well-defined, localized hyperdensity surrounded by hypodensity in the right body of the mandible in 45 and 46 regions (Figure 4). It extended antero-posteriorly from the distal aspect of 43 to the mesial aspect of 48. Supero-inferiorly from the cervical third of the root of 43 to the inferior border of the mandible, measuring approximately 33.7 mm x 33.3 mm in its maximum dimension (Figure 4). Its border was well-defined with a soft tissue outline. The lesion showed foci of internal calcifications, appearing as small hyperdense islands of bone areas within the soft-tissue mass, which coalesced and were suggestive of osseous/cementoid material. The alveolar cortical bone appeared intact, with no evidence of cortical perforation or destruction. Mild superficial pressure remodeling or shallow cupping of the crestal alveolar bone was seen adjacent to the lesion. There was no cortical breach. Surrounding trabecular bone showed a normal pattern. The radiographic diagnosis of the lesion was reported as a peripheral ossifying fibroma.

**Figure 4:**
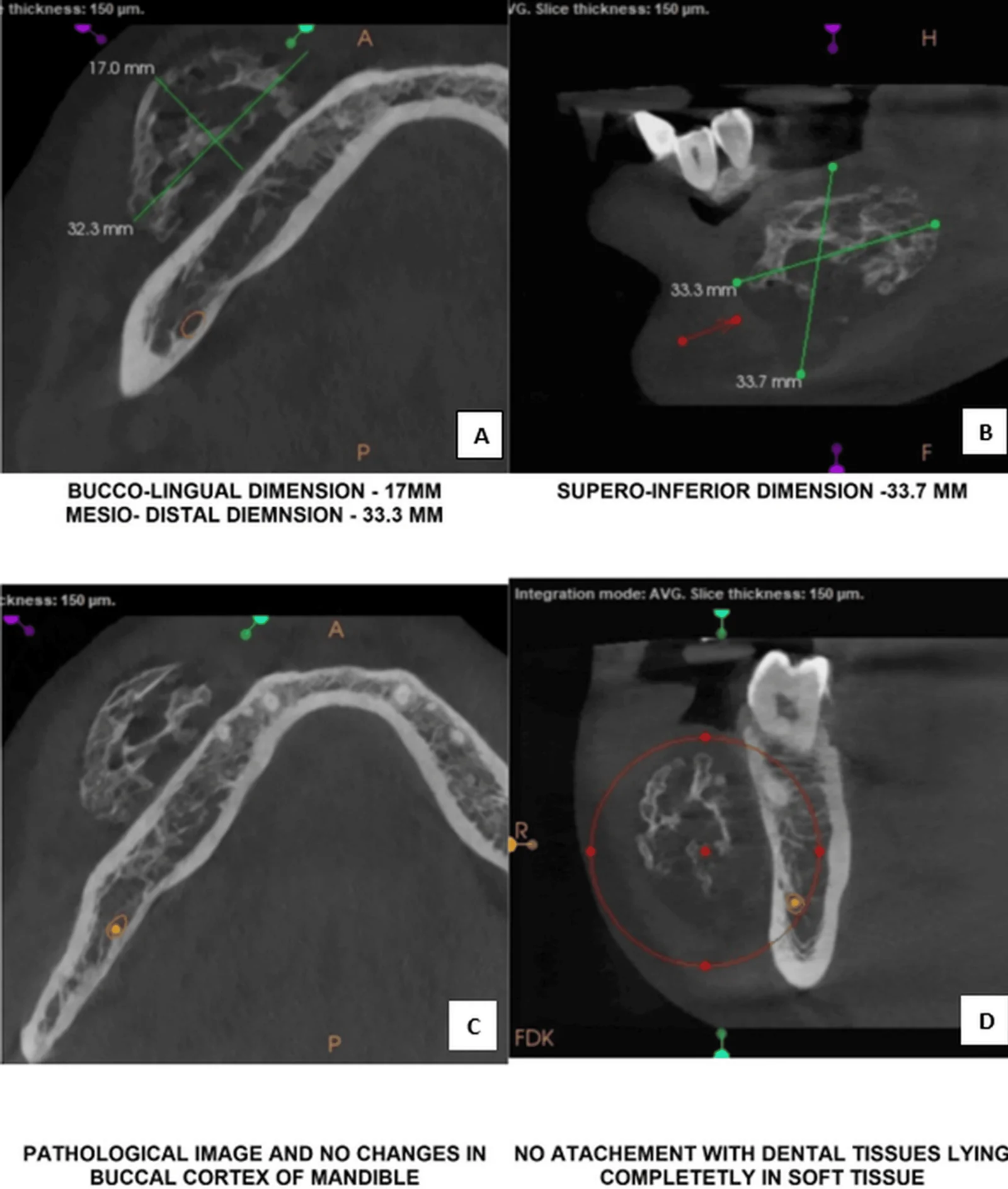
Cone beam computed tomography images A: Axial section, which showed the buccolingual dimension of 17 mm and mesiodistal dimension of approx. 33.3 mm in the right mandibular region. B: Sagittal section view demonstrating a well-defined lesion with a supero-inferior extent of approx. 33.7 mm at its greatest dimension. C: Axial section showed no evident alteration or perforation of the buccal cortical plate of the mandible. D: Coronal section showed that the lesion was completely confined to the soft tissue with no attachment or continuity with adjacent dental structures.

An excisional biopsy was performed by the department of Oral and Maxillofacial Surgery under general anesthesia (Figure 5). Following the biopsy, a radiograph of the specimen was taken (Figure 6).

**Figure 5:**
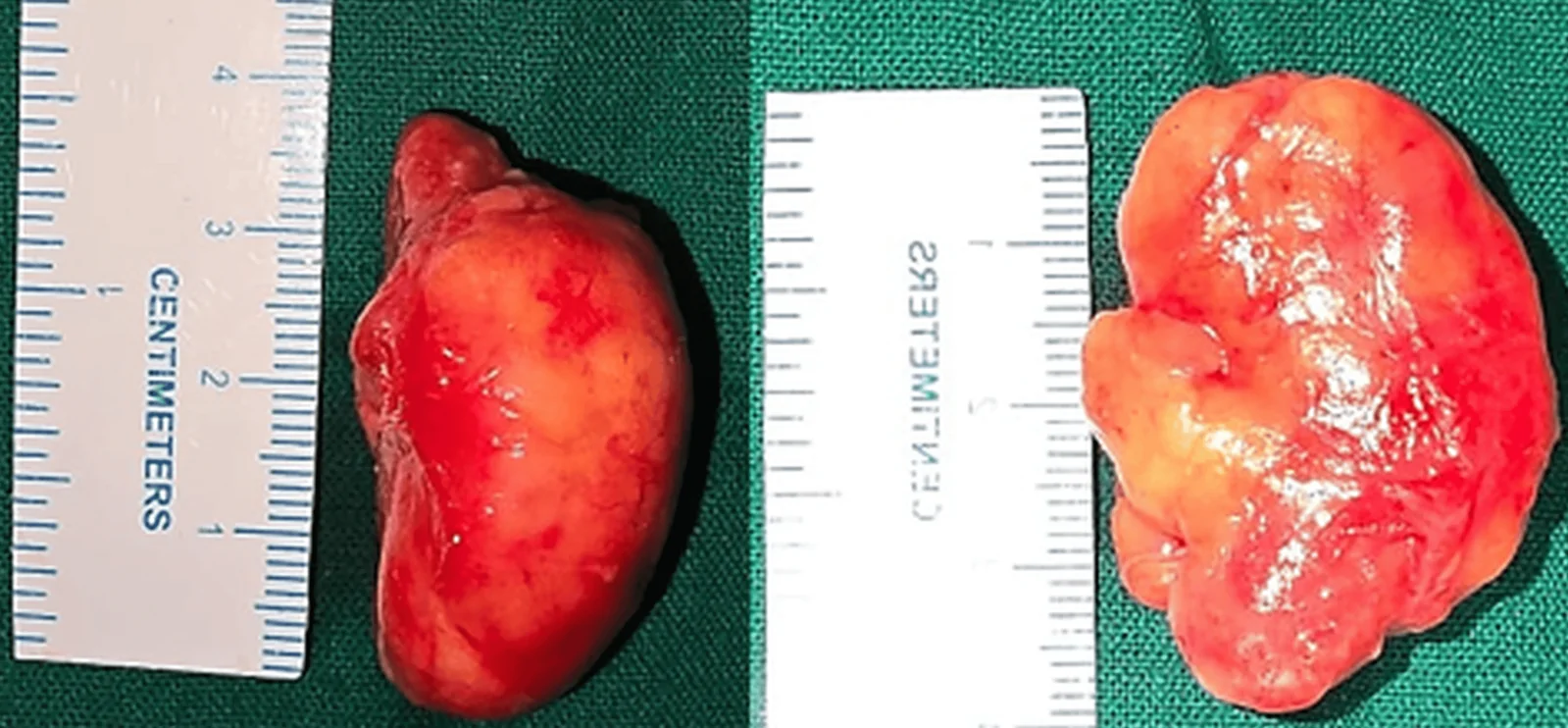
Excisional biopsy specimen

**Figure 6:**
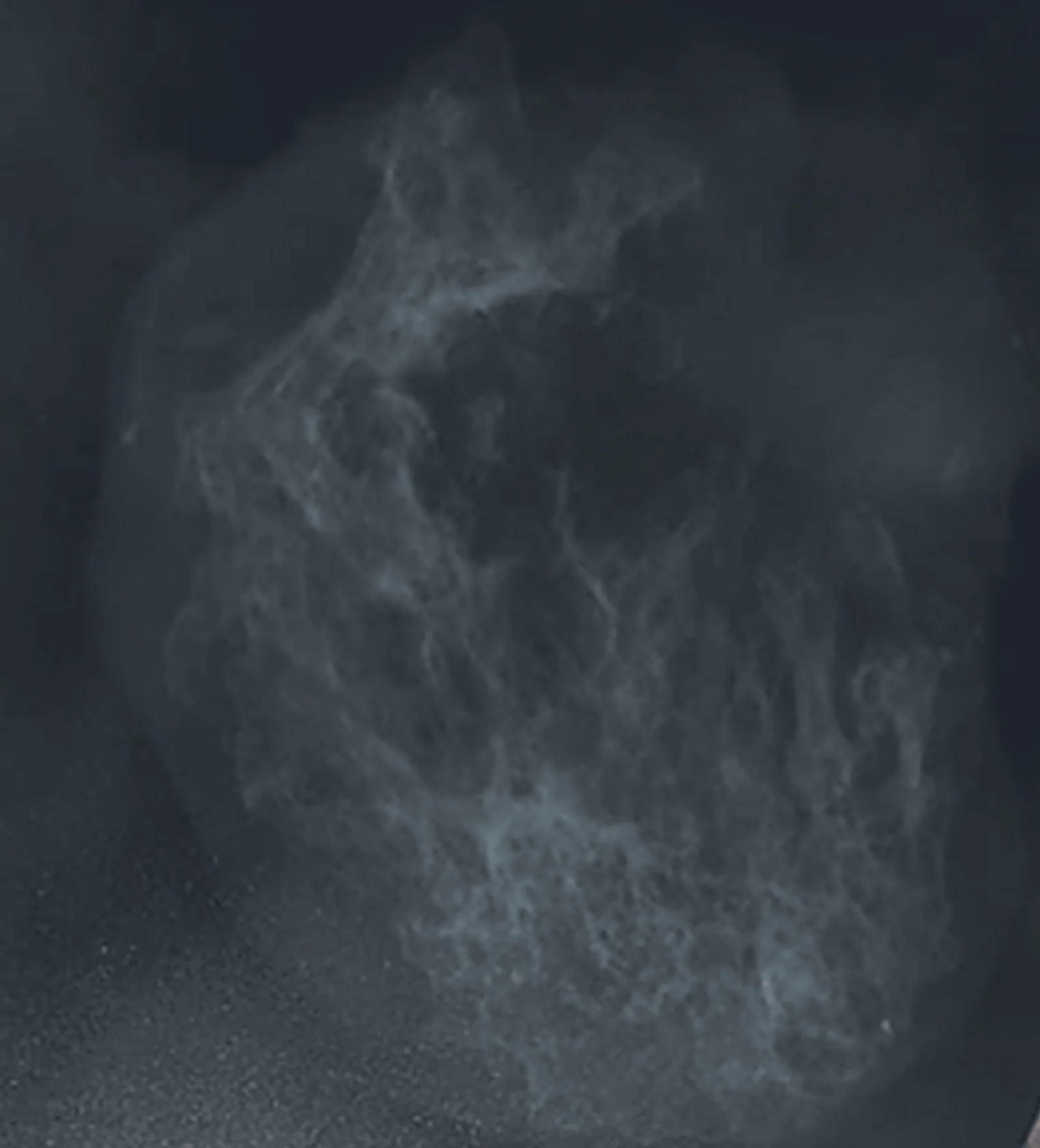
Postoperative radiograph of the specimen

Histopathological examination using H and E stain revealed an encapsulated lesion composed of mature adipose tissue interspersed with bony trabeculae. Adipocytes were observed, arranged in lobules separated by dense fibrous septae. There were areas of ossification, osteoid, and trabeculae of the bone with osteoid rimming and prominent resting lines seen within the adipose tissue (Figure 7). Focal areas with increased stromal cellularity were also observed surrounding the bony trabeculae with areas of osseous metaplasia (Figure 8). Based on this, a final report of osteolipoma was given.

**Figure 7:**
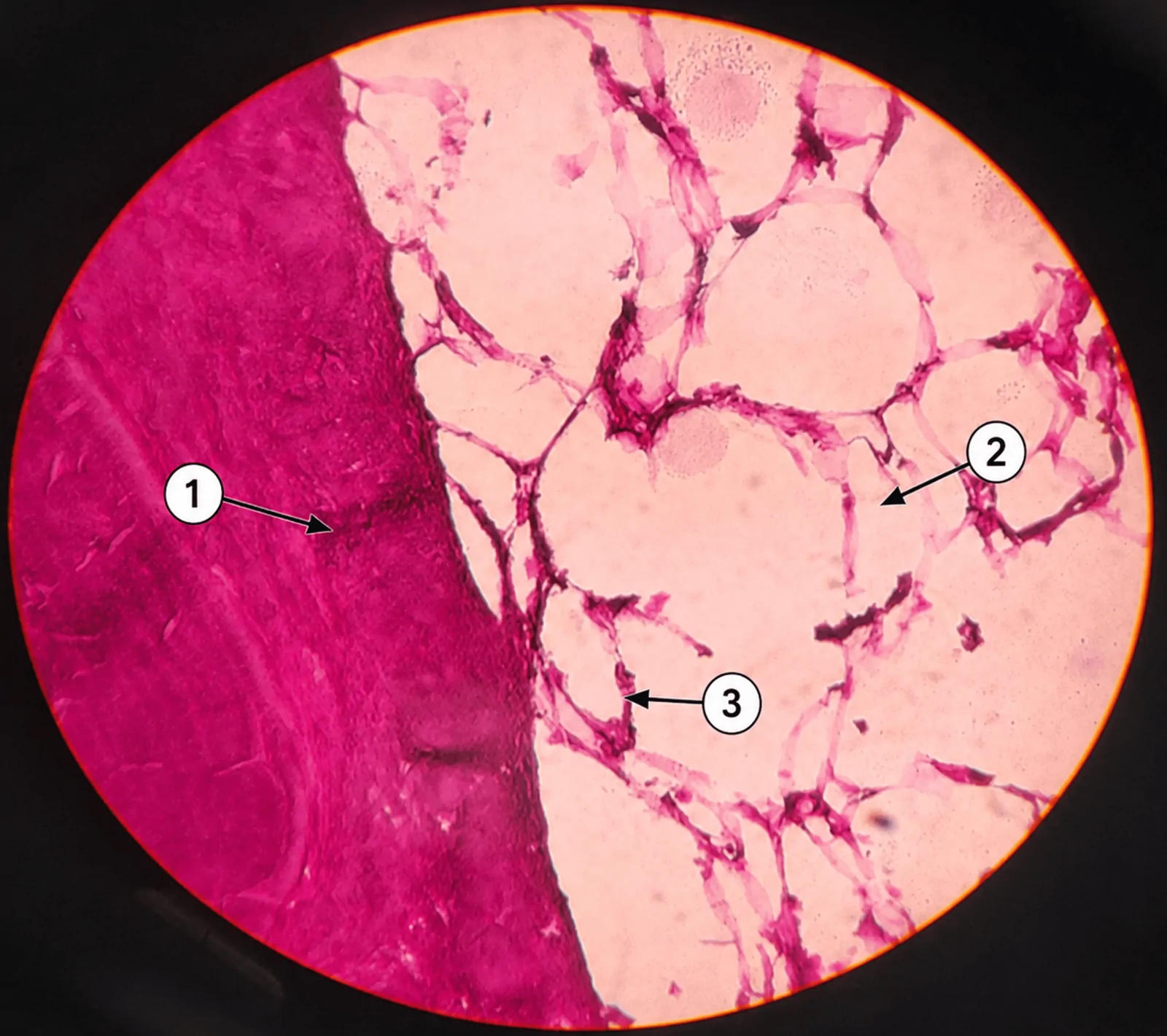
Photomicrograph at x40 magnification (hematoxylin and eosin stain) 1: Mature lamellar bone. 2: Mature adipocyte. 3: Fibrous septa/connective tissue

**Figure 8:**
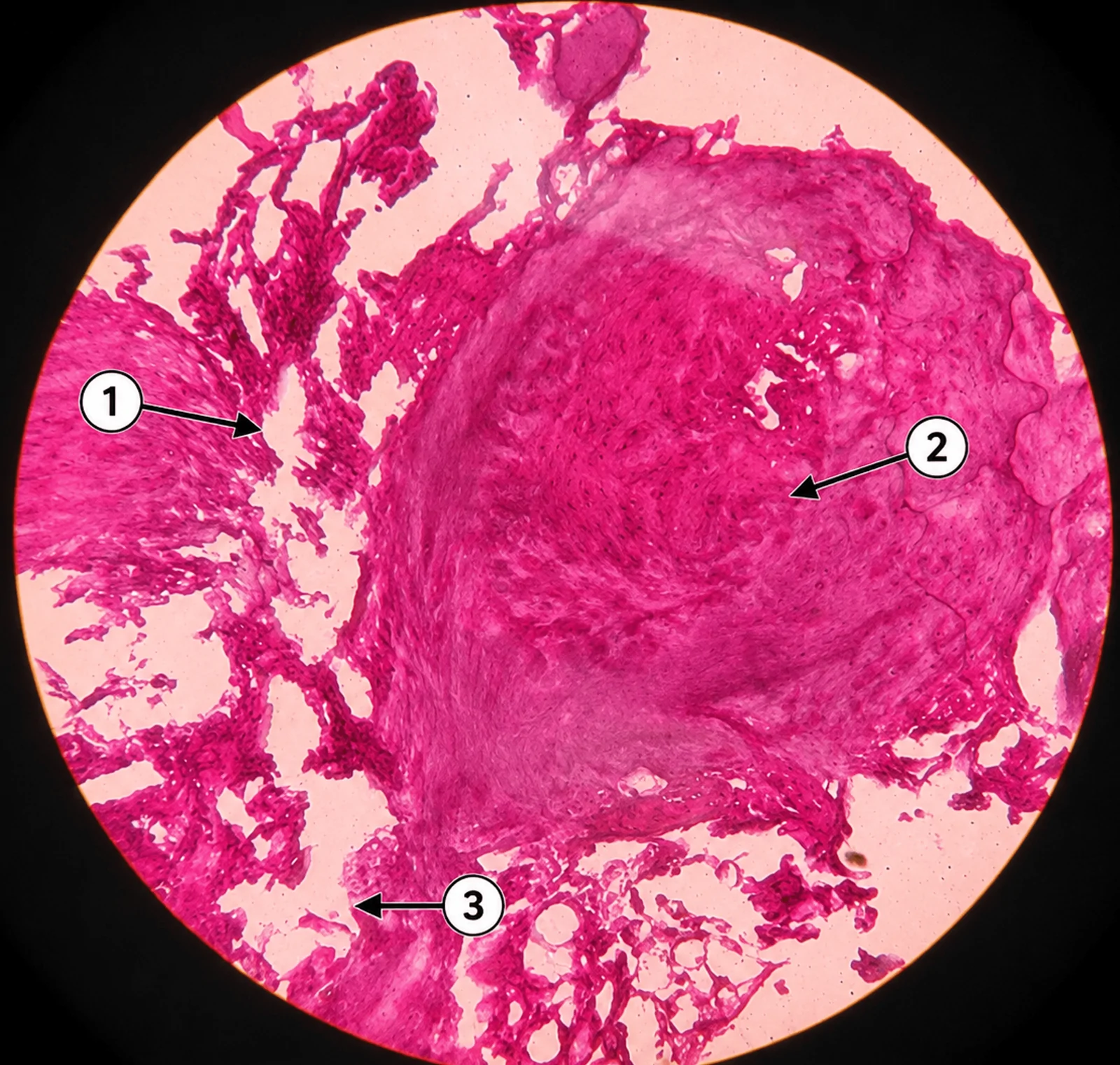
Photomicrograph at x40 magnification (hematoxylin and eosin stain) 1: Trabeculae of mature lamellar bone. 2: Osteoid (woven) bone. 3: Marrow spaces with adipocytes

## Discussion

A lipoma is a common benign mesenchymal tumour that can occur anywhere in the body. However, its occurrence in the oral cavity is relatively uncommon, accounting for approximately 1-4% of all benign oral soft-tissue tumours, as reported by Neville et al. and Furlong et al. [1,2]. Several histopathological variants of lipoma have been described, including fibrolipoma, angiolipoma, spindle cell lipoma, chondrolipoma, and osteolipoma, as detailed by Weiss et al. [3]. Among these, osteolipoma is an exceptionally rare variant characterized by mature adipose tissue admixed with well-formed lamellar bone [4].

Osteolipoma has been reported across a wide age range (6-81), most commonly in middle-aged adults, with no consistent gender predilection [6-8]. The present case occurred in a 40-year-old male patient, which is in accordance with the previously reported observations.

Clinically, osteolipomas usually present as slow-growing, painless, well-circumscribed masses, closely resembling conventional lipomas. Similar clinical presentations have been described [5]. The firm to hard consistency observed in some cases, including the present one, is attributed to the presence of ossified components within the lesion. Facial asymmetry and vestibular obliteration, as observed in this patient, have also been reported in larger lesions [8-10].

Radiographically, osteolipomas show variable appearances depending on the degree of osseous differentiation. While conventional lipomas are radiolucent, osteolipomas often present as mixed radiolucent-radiopaque lesions with internal calcifications. Similar radiographic findings and diagnostic dilemmas have been documented by various authors, where osteolipomas were initially misdiagnosed as fibro-osseous lesions or peripheral ossifying fibromas [8]. In the present case, CBCT findings of internal calcifications led to a provisional diagnosis of peripheral ossifying fibroma, consistent with these reports.

The exact pathogenesis of osteolipoma remains unclear. Osseous metaplasia within a pre-existing lipoma induced by chronic irritation, ischemia, or trauma was proposed by Katzer [6]. Alternatively, differentiation of pluripotent mesenchymal stem cells into adipocytes and osteoblasts has been suggested. The presence of mature adipose tissue with trabeculae of lamellar bone and areas of osseous metaplasia in the present case aligns with the metaplastic theory.

The differential diagnosis for this mixed radiolucent-radiopaque lesion included peripheral ossifying fibroma, myositis ossificans, chondrolipoma, osteochondroma, and well-differentiated liposarcoma, each distinguishable on clinical, radiographic, and histopathological features. Peripheral ossifying fibroma typically arises as a reactive gingival lesion associated with local irritants, showing scattered radiopaque flecks without a true capsule, whereas the present lesion was a submucosal, encapsulated mass unrelated to gingival irritation, with a fibroblastic stroma on histology that lacks mature adipocytic lobules. Ossifying fibroma presents as a hard, expansile intraosseous mass with a well-defined radiolucent rim, in contrast to the firm-to-soft consistency and diffuse mixed-density pattern seen here, and its cellular fibrous stroma similarly lacks an adipocytic component. Myositis ossificans usually follows a history of trauma and shows a zonal radiographic pattern with peripheral ossification and a central lucent zone within muscle, differing from the absence of trauma and the uniform admixture of fat and bone seen throughout the present lesion. Chondrolipoma may mimic osteolipoma clinically but shows flocculent, ring-like calcifications typical of cartilage matrix on imaging and lacks the trabecular bone and osteoblastic rimming identified histologically in this case. Osteochondroma classically arises from the condyle as an exophytic growth in bony continuity with the underlying cortex and medullary cavity, producing progressive facial asymmetry and malocclusion, unlike the discrete, mobile, non-bone-attached swelling seen in this patient. Well-differentiated liposarcoma with osseous metaplasia, the principal malignant consideration, tends to be larger and more rapidly growing with infiltrative, poorly defined margins radiographically, and shows nuclear atypia, lipoblasts, and mitotic activity histologically, all absent in the present, well-circumscribed, cytologically bland lesion. Therefore, careful correlation of clinical, radiographic, and histopathological findings was essential to establish the diagnosis of osteolipoma.

Complete surgical excision is the treatment of choice, and recurrence is exceedingly rare. Excellent prognosis following excision has been reported in previous case studies. The present case was successfully managed with complete excision and will be followed up for chances of recurrence, if any, though not reported to date.

Histopathological examination remains the gold standard for definitive diagnosis. Characteristic features include an encapsulated lesion composed of lobules of mature adipocytes separated by fibrous septae and interspersed with lamellar bone, osteoid formation, and osteoblastic rimming without cellular atypia, as consistently reported in the literature [4,5,9].

Due to its rarity and tendency to mimic other benign and fibro-osseous lesions, reporting additional cases of oral osteolipoma, as emphasized by Wong et al. [11], is essential to broaden understanding of its clinicopathological spectrum and biological behavior.

In the oral and maxillofacial region, osteolipomas have been described at various anatomical sites. Piattelli et al. reported involvement of the tongue, while Firth et al. and de Castro et al. documented cases in the buccal mucosa [4,9,12]. Lesions involving the palate have been reported by Adebiyi et al. [13]. Mandibular involvement, as seen in the present case, is distinctly rare and has only been reported twice in the literature, notably by Karabas et al. [14].

## Conclusions

Osteolipoma of the oral cavity is an exceedingly rare benign lesion, and its key clinical significance lies in its ability to closely mimic fibro-osseous and other calcified lesions on both clinical and radiographic examination. In the present case, complete surgical excision resulted in uneventful healing with no evidence of recurrence at follow-up, underscoring excision as a curative treatment with an excellent prognosis. While clinical assessment and imaging help narrow the differential diagnosis, histopathological examination remains essential for a definitive diagnosis. Documentation of such rare cases contributes to improved diagnostic awareness and understanding of this uncommon entity.
